# Dietary patterns and osteoporosis risk in a high-altitude population: the roles of gut microbiota and plasma metabolites in a case-control study

**DOI:** 10.3389/fnut.2026.1865666

**Published:** 2026-09-10

**Authors:** Luhan Zhang, Xiaomin Wu, Xuan Chen, Haihong Xin, Jingang Yan, Majia Zhuo, Xiaoxia Ma, Xiaozhi Liu, Mingyong Liu, Guowei Huang, Fei Ma

**Affiliations:** 1 Department of Epidemiology and Biostatistics, School of Public Health, Tianjin Medical University, Tianjin, China; 2 Key Laboratory of Prevention and Control of Major Diseases in the Population, Ministry of Education, Tianjin Medical University, Tianjin, China; 3 Tianjin Key Laboratory of Environment, Nutrition and Public Health, Tianjin, China; 4 Department of Orthopedics, Huangnan Tibetan Autonomous Prefecture People’s Hospital, Huangnan Tibetan Autonomous Prefecture, Qinghai, China; 5 Department of Nursing, Huangnan Tibetan Autonomous Prefecture People’s Hospital, Huangnan Tibetan Autonomous Prefecture, Qinghai, China; 6 Tianjin Key Laboratory of Epigenetics in Premature Infant Organ Development, Tianjin Fifth Central Hospital, Tianjin, China; 7 Tianjin Binhai Huangnan Plateau Medical Research Institute, Huangnan Tibetan Autonomous Prefecture People’s Hospital, Huangnan Tibetan Autonomous Prefecture, Qinghai, China; 8 Emergency Medicine Research Institute, Tianjin Fifth Central Hospital, Tianjin, China; 9 Department of Nutrition and Food Science, School of Public Health, Tianjin Medical University, Tianjin, China

**Keywords:** dietary patterns, gut microbiota, high-altitude regions, osteoporosis, untargeted metabolomics

## Abstract

**Background:**

This study investigated the association between dietary pattern and osteoporosis, exploring gut microbiota and plasma metabolites as potential mediators.

**Methods:**

This case-control study included 90 osteoporosis patients and 90 healthy controls. All participants completed a validated semi-quantitative food frequency questionnaire (FFQ) to assess their dietary intake over the past year. Principal component analysis (PCA) was performed on the FFQ data to identify major dietary patterns. Gut microbiota composition was analyzed using metagenomic sequencing (Illumina HiSeq platform, PE150). Plasma metabolites were analyzed using untargeted metabolomics with ultra-high-performance liquid chromatography-tandem mass spectrometry (UHPLC-MS/MS). Binary logistic regression was employed to examine the association between dietary pattern and osteoporosis; Multivariate linear regression was used to investigate the association between dietary pattern and gut microbiota/metabolites. Additionally, mediation analysis was conducted to assess whether gut microbiota or plasma metabolites mediated the association between dietary patterns and osteoporosis, and the proportion of mediation was calculated.

**Results:**

The osteoporosis group showed significant differences in age (61.89 ± 7.86 vs. 56.10 ± 7.29 years), sex (72.2% vs. 36.7% female), and anthropometrics (all *P* < 0.05). Among six identified dietary patterns, “animal offal and seeds” was associated with higher osteoporosis risk (OR = 2.21, 1.21–4.06; *P* = 0.010). osteoporosis patients exhibited gut microbiota dysbiosis (enriched Enterobacteriaceae) and seven metabolites exerted mediating effects on the association between dietary pattern and osteoporosis.

**Conclusion:**

In high-altitude population, the “animal offal and seeds” dietary pattern elevates osteoporosis risk, partially mediated by metabolite. Gut microbiota composition also differs significantly between osteoporosis patients and controls.

## 1 Introduction

Osteoporosis is a systemic skeletal disorder characterized by reduced bone mass and deterioration of bone microarchitecture, resulting in increased bone fragility and a significantly higher risk of fractures. This condition predominantly affects postmenopausal women and elderly men (1). With the aging global population, osteoporosis has become an increasingly critical public health issue. Epidemiological studies indicate that approximately 21.7% of older adults worldwide are affected by this disease, with the highest prevalence (24.3%) reported among the elderly in Asia (2, 3). A study examining osteoporosis prevalence across different altitude zones in Sichuan Province, China, found an overall prevalence rate of 19.42% among adults aged 50 and above. Notably, the medium-altitude region (average altitude: 588 m) exhibited a significantly higher prevalence rate of 25.85%, surpassing both the national average for Chinese adults in the same age group. In contrast, the high-altitude region (average altitude: 2,500–3,000 m) demonstrated a lower prevalence of 13.00% (4). This underscores the urgent need to strengthen early screening, standardized management, and effective interventions to mitigate the burden and socioeconomic impact of osteoporosis.

Bone, as a metabolically active tissue, is closely linked to nutritional factors crucial for maintaining skeletal health. Throughout life, various nutrients work synergistically in bone formation, remodeling, and homeostasis, playing a pivotal role in preserving bone integrity. However, traditional single-nutrient research approaches have notable limitations. These approaches fail to capture the complex interactions among multiple nutrients *in vivo* and struggle to explain the heterogeneity observed across different studies. In contrast, dietary pattern analysis, which integrates nutrient combinations and their interactions within the overall dietary structure, provides a more systematic assessment of diet’s impact on bone health (5). This approach offers a novel perspective that addresses the limitations of single-nutrient research.

The gut microbiota, as the largest microbial ecosystem in the human body, forms a dynamic and balanced symbiotic relationship with the host. This microbial community undergoes adaptive evolution in response to host development and environmental changes, establishing a bidirectional regulatory mechanism. Dysbiosis of the microbiota can significantly impair host health (6). Recent studies have revealed that gut microbiota profoundly influences bone metabolism through two key pathways: immune regulation and metabolic modulation. Microbial metabolites, such as short-chain fatty acids, indole derivatives, and secondary bile acids, bind to immune cell surface receptors (e.g., GPR43, aryl hydrocarbon receptor), regulating T-cell differentiation (particularly the dynamic balance between regulatory T cells and Th17 cells) and modulating osteoclast activity and bone resorption (7, 8). Dietary nutrition plays a crucial role in shaping the gut microbiota, with different dietary patterns significantly altering its composition and functional characteristics. Research has shown that high intake of monounsaturated fatty acids (MUFA) is associated with a reduced abundance of Bifidobacterium and a slight increase in Bacteroides levels, while excessive consumption of ω-6 polyunsaturated fatty acids (PUFAs) leads to a decline in Bifidobacterium population. In contrast, a fiber-rich diet selectively promotes the growth of beneficial short-chain fatty acid-producing bacteria (such as Bifidobacterium and Roseburia), while suppressing the growth of potential pathogens (9).

Gut microbiota and their co-metabolic bioactive molecules can cross the intestinal mucosal barrier and enter systemic circulation. Plasma concentrations of these molecules have been shown to significantly correlate with the metabolic activity of gut microbiota (10). This crucial finding suggests that plasma metabolites can serve as “distal biomarkers” reflecting the functional output of gut microbes, offering a unique perspective to explore microbiota-host interactions and their roles in disease pathogenesis. By employing highly sensitive blood metabolomics technologies, researchers can track the metabolic impacts of dietary and other environmental factors on the host, while systematically monitoring changes in gut microbial metabolic profiles. This approach provides a powerful tool for in-depth analysis of complex interaction networks (11).

Most current research on dietary factors and osteoporosis has focused on population in plain areas, while studies in high-altitude regions remain largely unexplored. Huangnan Tibetan Autonomous Prefecture lies in the southeastern Qinghai-Tibet Plateau. It is a multi-ethnic settlement dominated by Tibetan residents, where the regional altitude mostly exceeds 3,000 m. Restricted by local high-altitude surroundings and traditional ethnic living customs, local residents consume large amounts of meat yet insufficient vegetables and fruits. Their habitual diet features high-oil, high-salt, high-protein and high-cholesterol intake alongside low vitamin intake. The distinctive ethnic-related dietary characteristics among residents in the Qinghai-Tibet Plateau provide an ideal model for investigating the relationship between diet and osteoporosis. Therefore, this study conducted a hospital-based case-control study in the high-altitude region of Huangnan Tibetan Autonomous Prefecture, Qinghai Province, aiming to explore dietary pattern associated with osteoporosis. Using metagenomic sequencing and untargeted metabolomics technologies, this study systematically analyzed differences in gut microbiota composition and metabolic profiles, further investigating the role of gut microbes and metabolites in the association between dietary pattern and osteoporosis.

## 2 Methods

This hospital-based case-control study was conducted at the Department of Orthopedics, Huangnan Tibetan Autonomous Prefecture People’s Hospital, Qinghai Province, from July to October 2024. Participants were permanent residents of Qinghai Province aged 50 years and older. The study protocol was approved by the Institutional Review Board of Huangnan Tibetan Autonomous Prefecture People’s Hospital (Approval No. HNH20240902), and all participants provided written informed consent. The technical roadmap is shown in Supplementary Figure 1.

### 2.1 Study subjects

#### 2.1.1 Diagnostic criteria for osteoporosis

Osteoporosis cases were diagnosed with reference to the World Health Organization (WHO) diagnostic criteria for osteoporosis (12), combined with bone-mineral-density test results detected by the BMD-9 quantitative ultrasound bone densitometer (Yueqi Medical, Beijing, China) and clinical medical history, defined by meeting any of the following criteria: (1) hip or vertebral fragility fractures; (2) bone mineral density (BMD) T-score ≤ −2.5 at the axial skeleton or distal 1/3 radius; (3) osteopenia (−2.5 < T-score < −1.0) combined with fragility fractures at the proximal humerus, pelvis, or distal forearm, while excluding recent use of medications affecting bone metabolism and endocrine or immune disorders influencing bone metabolism. The control group consisted of non-osteoporotic residents with normal BMD values.

#### 2.1.2 Sample size estimation

This study employed an unmatched case-control design. The sample size was calculated using PASS software (Version 21.0). Based on prior literature, the prevalence of high-fat diet exposure in the population was 40.1%, with an expected odds ratio (OR) of 2.77, a significance level (α) of 0.05, and 90% statistical power (13, 14). The calculation indicated a required sample size of 80 subjects per group. To account for potential participant attrition, 90 cases and 90 controls were ultimately enrolled.

### 2.2 Questionnaire survey

Sociodemographic characteristics (age, sex, ethnicity, education level, marital status), lifestyle factors (smoking, alcohol consumption, physical activity, sleep patterns), and anthropometric measurements (height, weight, waist circumference, hip circumference) were collected via face-to-face interviews.

This study employed standardized methods for physical activity data processing. First, all activity durations were converted to minutes, and individuals with missing data were excluded. A maximum daily activity duration of 960 min (16 h) was set, with exceeding cases being excluded, while single activity episodes lasting less than 10 min were recorded as 0. For short-form questionnaire data, truncation rules were applied: daily activity duration of the same intensity exceeding 180 min was truncated to 180 min, with a weekly upper limit of 1,260 min for each intensity level. Finally, based on the processed data, participants were categorized into low, medium, and high physical activity level groups according to predefined criteria (15).

Smoking was classified as: (1) Never smokers (“smoking status” = no); (2) Current smokers (“smoking status” = yes and “years since quitting” = 0); (3) Former smokers (“smoking status” = yes and “years since quitting” > 0). Alcohol consumption was categorized as: (1) Never drinkers (“alcohol consumption” = no); (2) Current drinkers (“alcohol consumption” = yes and “years since quitting alcohol” = 0); (3) Former drinkers (“alcohol consumption” = yes and “years since quitting alcohol” > 0).

### 2.3 Bone mineral density testing

Bone mineral density at the distal 1/3 radius of participants was measured using a quantitative ultrasound bone densitometer (BMD-9M). The primary reported parameter was speed of sound (SOS), with the device software automatically calculating evaluation indices, including T-scores and Z-scores, based on reference population data.

During testing, participants sat upright facing the device, with the measured arm naturally positioned on the examination platform, fully exposing the distal 1/3 radial region after removing all accessories. Participants maintained natural arm extension with palms facing downward, wrists in a neutral position, and torso erect, while avoiding muscle tension in the arms. After entering the required participant information, the operator initiated the testing procedure, which concluded when the device signaled completion. Participants were required to remain completely still during the measurement. The Asian population database was used as the reference standard for parameter calculation.

### 2.4 Dietary assessment

A semi-quantitative food frequency questionnaire (FFQ) was used to assess participants’ dietary intake over the past year. The FFQ was adapted to include region-specific food items in Qinghai, with detailed inquiries on consumption frequency and average portion sizes for each food category. Seasoning intake (salt, cooking oil) was estimated based on household consumption and the number of diners.

### 2.5 Metagenomic sequencing

Gut-microbiota profiles were detected via metagenomic sequencing. Fresh morning fecal specimens (3–5 g) were collected under sterile conditions, transported at low temperature and preserved at −80 °C. Fecal microbial DNA was extracted and qualified for concentration and integrity with Qubit and Qsep400. Qualified DNA was enzymatically sheared into ∼300-bp fragments before end-repair, A-tailing, adapter ligation, purification and PCR-based library amplification. Libraries were assayed by Qubit 4.0 and Qsep400 for concentration and insert-size screening. Eligible libraries were pooled and subjected to Illumina PE150 paired-end sequencing. Raw reads were trimmed to remove adapters and low-quality sequences to generate clean data. Individual-sample *de novo* assembly and mixed assembly of unassembled reads from all samples were performed to capture low-abundance microbes. After contig screening, CDS prediction and sequence clustering, non-redundant Unigenes and their abundance matrix were yielded. Taxonomic annotation from kingdom to species level was accomplished against the MicroNR database. Rarefaction curves, species-accumulation boxplots and rank-abundance curves assessed sequencing-depth sufficiency. Alpha-diversity metrics (Observed species, Chao1, Shannon) were calculated. Bray-Curtis-distance-based PCoA and NMDS illustrated inter-group microbial-community dissimilarity. The top-10 most-abundant taxa at each taxonomic rank were reserved and the rest merged into “Others.” Linear discriminant analysis Effect Size (LEfSe) analysis screened differential microbial biomarkers, with LDA scores adopted to measure biomarker effect-size.

### 2.6 Metabolomics analysis

Fasting venous blood samples (5 mL for each subject) were centrifuged at 4 °C (3,000 × *g*, 10 min) to isolate plasma, which was aliquoted and stored at −80 °C. Untargeted metabolomic analysis was performed using liquid chromatography-tandem mass spectrometry (LC-MS/MS). Raw data were converted to mzML format via ProteoWizard, followed by peak picking and alignment using XCMS. Missing values were imputed using k-nearest neighbors (kNN), and signal drift was corrected by support vector regression (SVR). Metabolites were identified by matching against HMDB/METLIN databases and MS/MS spectra, with data from both positive and negative ion modes merged (CV < 0.3). Probabilistic quotient normalization (PQN) was applied before downstream analyses. The untargeted metabolomic profiling was carried out with a QExactiveHF-X mass spectrometer and Vanquish ultra-high-performance liquid-chromatography system (Thermo Scientific, USA).

### 2.7 Statistical analysis

Data processing and statistical analysis were performed using SPSS 27.0 and R 4.4.2 software. Continuous variables with normal distribution were expressed as mean ± standard deviation (Mean ± SD) and compared using the independent samples *t*-test, while non-normally distributed continuous variables were presented as median (interquartile range, IQR) and analyzed using the Mann-Whitney U test. Categorical variables were presented as frequency [percentage, *n* (%)], and group differences were evaluated using the chi-square test (χ^2^ test).

Dietary patterns were extracted using exploratory factor analysis (EFA), with data suitability verified by the Kaiser-Meyer-Olkin (KMO) test (KMO > 0.6) and Bartlett’s test of sphericity (*P* < 0.05). Binary logistic regression was used to analyze the association between dietary patterns and osteoporosis.

Differential microbial taxa were analyzed based on the NCBI RefSeq database, and functional pathways were annotated using the Kyoto Encyclopedia of Genes and Genomes (KEGG) database. Differential metabolites were screened by combining multivariate analysis (e.g., partial least squares-discriminant analysis, PLS-DA) and univariate analysis (e.g., *t*-test). To explore the relationship between gut microbiota/metabolomic biomarkers and osteoporosis or dietary patterns, fully adjusted models (accounting for covariates such as age and sex) were constructed. Binary logistic regression (for categorical variables) or multiple linear regression (for continuous variables) was applied. Multiple testing corrections were performed using the Benjamini-Hochberg method (FDR < 0.05). Causal mediation analysis based on regression was conducted to assess the potential mediating role of gut microbiota/metabolomic biomarkers in the diet-osteoporosis association. The (interquartile range, IQR) and the proportion of the total effect (PIE) were calculated for each candidate mediator. All analyses were two-sided, with a significance threshold set at α = 0.05.

## 3 Results

### 3.1 General characteristics

The demographic characteristics and lifestyle factors of the osteoporosis case group and control group are summarized in Table 1. This study included a total of 180 participants, with 90 in each group. The mean age of participants in the osteoporosis group was significantly higher than that of the control group (61.89 ± 7.86 vs. 56.10 ± 7.29 years, *P* < 0.001), and the proportion of females was notably higher in the osteoporosis group (72.2% vs. 36.7%, *P* < 0.001). Regarding anthropometric measurements, the osteoporosis group had significantly lower values for both height [1.61 (1.55, 1.68) vs. 1.67 (1.60, 1.70) m, *P* < 0.001] and weight (67.96 ± 12.06 vs. 71.82 ± 10.67 kg, *P* = 0.024) compared to the control group. Additionally, the use of calcium supplements was significantly higher in the osteoporosis group (18.9% vs. 7.8%, *P* = 0.028). However, no significant statistical differences were found between the two groups regarding education level, ethnicity, marital status, smoking, alcohol consumption, body mass index (BMI), waist-to-hip ratio (WHR), physical activity level, sleep duration, difficulty falling asleep, snoring, or early awakening (all *P* > 0.05).

**TABLE 1:** Characteristics of study subjects.

Variables	Case (*n* = 90)	Control (*n* = 90)	*Z*/*t*/χ^2^	*P*
Age (year)	61.89 ± 7.86	56.10 ± 7.29	−4.880	<0.001
Sex			22.937	<0.001
Male	25 (27.8)	57 (63.3)	–	–
Female	65 (72.2)	33 (36.7)	–	–
Educational level			6.700	0.082
Illiterate	55 (61.1)	39 (43.3)	–	–
Primary school	25 (27.8)	31 (34.4)	–	–
Junior high school	4 (4.4)	8 (8.9)	–	–
High school or above	6 (6.7)	12 (13.3)	–	–
Ethnicity			0.390	0.530
Tibetan	57 (63.3)	61 (67.8)	–	–
Others	33 (36.7)	29 (32.2)	–	–
Marital status			0.535	0.465
Unmarried/divorced/widowed	21 (23.3)	17 (18.9)	–	–
Married/cohabitating	69 (76.7)	73 (81.1)	–	–
Smoking			3.130	0.209
Current smoker	7 (7.8)	14 (15.6)	–	–
Former smoker	8 (8.9)	10 (11.1)	–	–
Never smoked	75 (83.3)	66 (73.3)	–	–
Alcohol consumption			5.717	0.057
Current drinker	7 (7.8)	12 (13.3)	–	–
Former drinker	2 (2.2)	8 (8.9)	–	–
Never drinker	81 (90.0)	70 (77.8)	–	–
Height (m)^a^	1.61 (1.55, 1.68)	1.67 (1.60, 1.70)	−3.336	<0.001
Weight (kg)	67.96 ± 12.06	71.82 ± 10.67	−2.271	0.024
BMI (kg/m^2^)^a^	26.43 (23.49, 29.16)	25.93 (24.21, 28.76)	−0.143	0.886
WHR^a^	0.93 (0.88, 0.96)	0.94 (0.89, 0.98)	−1.888	0.059
Physical activity			0.559	0.756
Low	3 (3.3)	2 (2.2)	–	–
Moderate	20 (22.2)	17 (18.9)	–	–
High	67 (74.4)	71 (78.9)	–	–
Sleep duration (hours)			1.990	0.370
<6.5	13 (14.4)	20 (22.2)	–	–
6.5–8.0	52 (57.8)	45 (50.0)	–	–
>8.0	25 (27.8)	25 (27.8)	–	–
Difficulty falling asleep			0.024	0.876
Yes	32 (35.6)	31 (34.4)	–	–
No	58 (64.4)	59 (65.6)	–	–
Snoring			0.851	0.356
Yes	59 (65.6)	53 (58.9)	–	–
No	31 (34.4)	37 (41.1)	–	–
Early awakening			0.227	0.634
Yes	62 (68.9)	59 (65.6)	–	–
No	28 (31.1)	31 (34.4)	–	–
Ca supplements			4.808	0.028
Yes	17 (18.9)	7 (7.8)	–	–
No	73 (81.1)	83 (92.2)	–	–

BMI, body mass index; WHR, Waist-to-Hip Ratio. Values have been presented as mean ± SD for normally distributed continuous variables, median (IQR) for non-normally distributed continuous variables, and frequency (percentage) for categorical variables.

^a^The distribution of variables is not normal, using the Mann-Whitney U test.

### 3.2 Dietary patterns and osteoporosis

**FIGURE 1:**
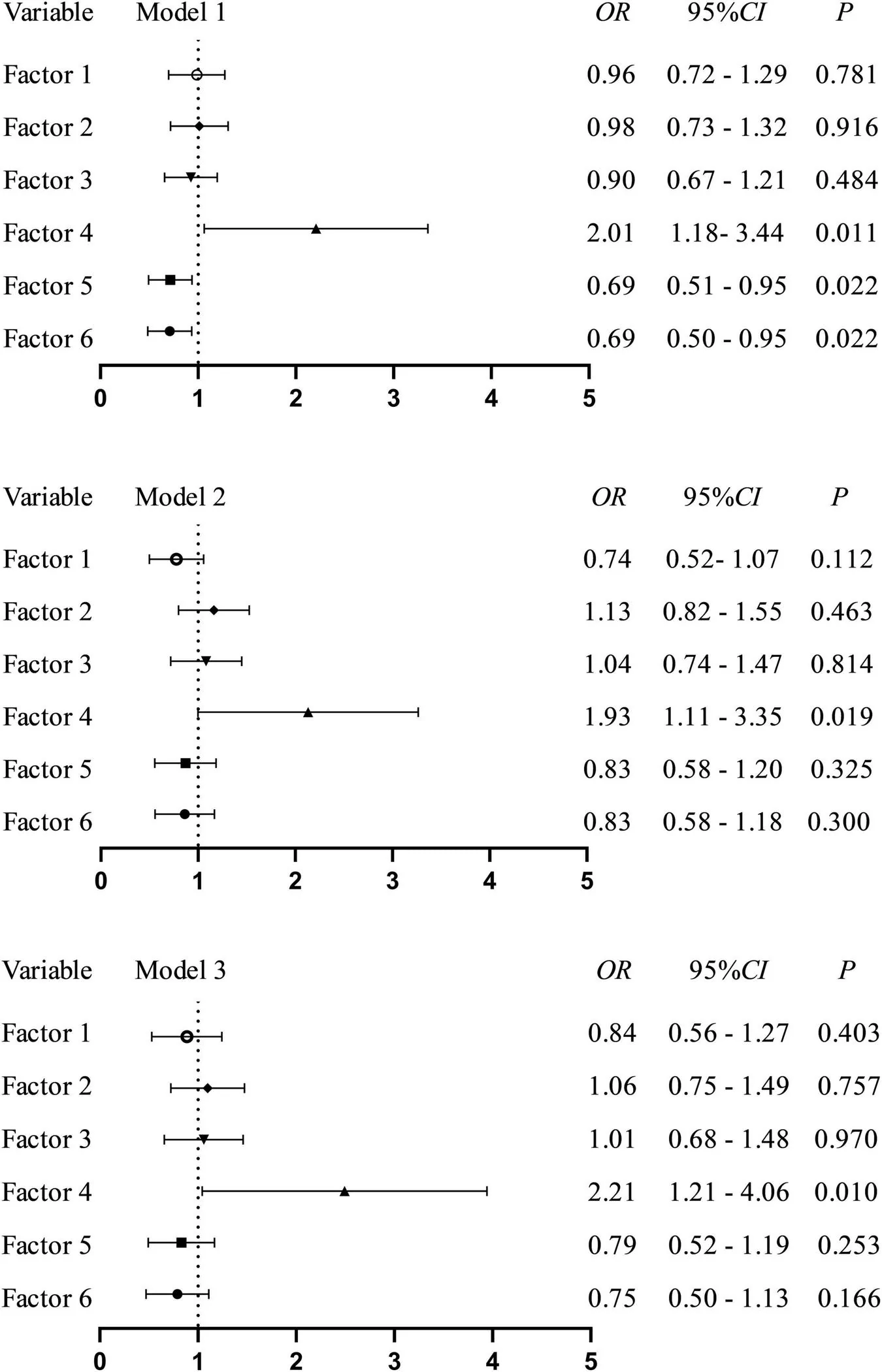
Association between dietary patterns and osteoporosis (OR, 95% CI). Model 1: unadjusted. Model 2: adjusted for age, sex, educational level. Model 3: adjusted for age, sex, educational level, smoking, alcohol consumption, Ca supplements, physical activity, height, weight, and waist-to-hip ratio (WHR).

Principal component factor analysis was conducted to identify major dietary patterns, resulting in six distinct patterns based on eigenvalues > 1: Factor 1, Plant-based Diet; Factor 2, High-Protein Seafood & Poultry Diet; Factor 3, Refined Grain-Dominant Diet; Factor 4, Animal Offal and Seeds Diet; Factor 5, High-Salt & High-Oil Diet; Factor 6, Red Meat-Based Diet (Supplementary Table 1). Binary logistic regression analysis revealed that the “animal offal and seeds” dietary pattern was significantly associated with an increased risk of osteoporosis (OR: 2.01, 95% CI: 1.18–3.44, *P* = 0.011). After adjusting for potential confounders, including age, sex, smoking, alcohol consumption, and physical activity level, the positive association remained statistically significant (OR: 2.21, 95% CI: 1.21–4.06, *P* = 0.010) (Figure 1).

### 3.3 The association between gut microbiota and osteoporosis

**FIGURE 2:**
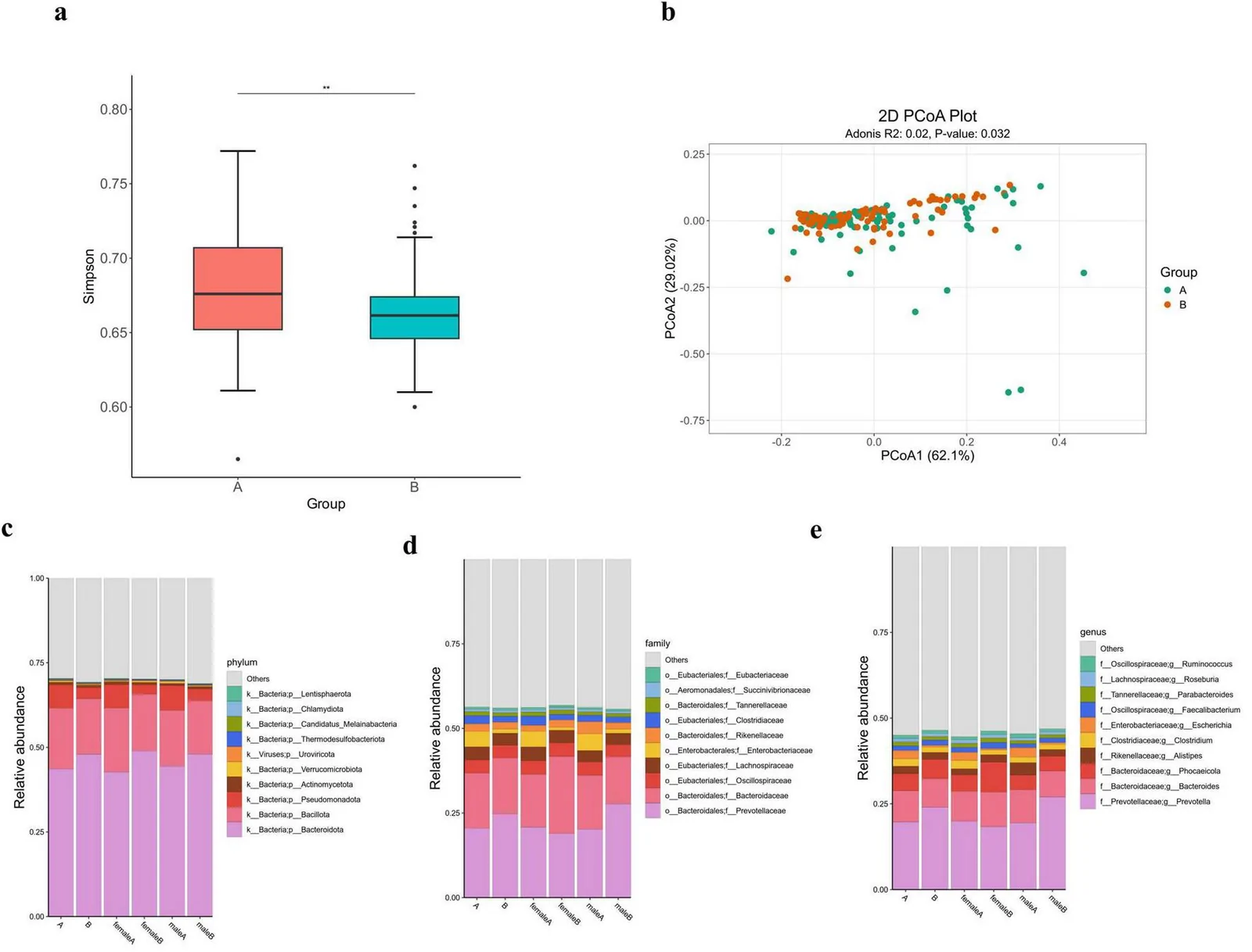
Gut microbiological characteristics. **(a)** α-diversity analysis - Simpson index. **(b)** Principal coordinate analysis (PCoA). **(c)** Relative abundance of gut microbiota at phylum level. **(d)** Relative abundance of gut microbiota at family level. **(e)** Relative abundance of gut microbiota at genus level.

Gut microbiome α diversity was significantly higher in the osteoporosis group compared to the control group. Specifically, the Simpson diversity index (1-D) was markedly elevated in the osteoporosis group (*P* < 0.01, Mann-Whitney U test) (Figure 2a). Additionally, β-diversity analysis based on Bray-Curtis distances (principal coordinate analysis, PCoA) confirmed distinct microbial community structures between the two groups (Figure 2b).

At the phylum level, the osteoporosis group displayed a different microbial composition, with 43.57% Bacteroidota and 18.02% Bacillota, leading to a significantly higher Firmicutes/Bacteroidota (F/B) ratio (41.36%). In contrast, the control group exhibited higher relative abundances of Bacteroidota (47.89%) and Bacillota (16.46%), resulting in a lower F/B ratio (34.37%) (Figure 2c). At the genus and family levels, the osteoporosis group showed reduced abundances of Prevotella and Bacteroides compared to the control group (Figures 2d, e).

**FIGURE 3:**
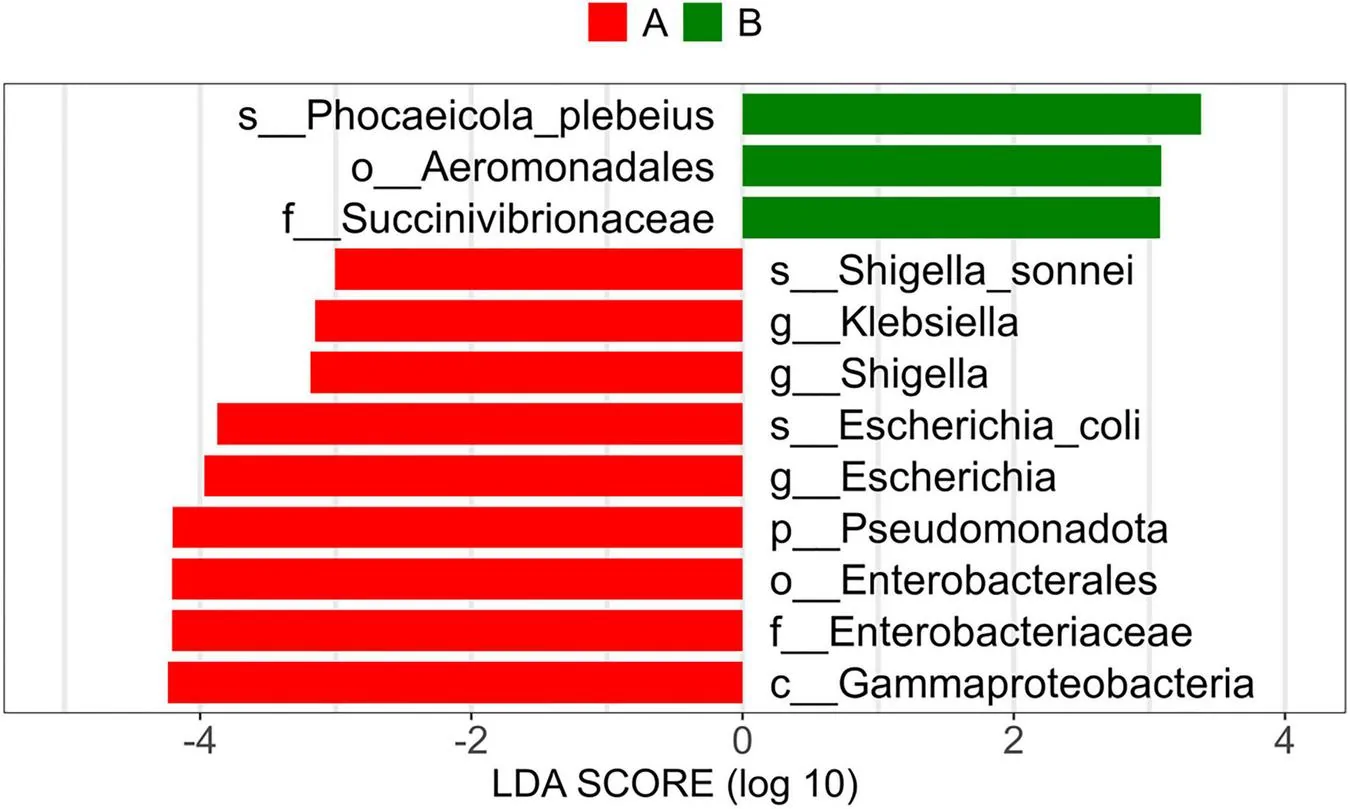
Histogram of the distribution of LDA values for the different species. A, osteoporosis group; B, control group.

LEfSe analysis of gut microbiome data (Figure 3 and Supplementary Table 2) revealed significant differences in microbial composition between the groups, with 12 taxa showing differential abundance (*P* < 0.05). The osteoporosis group exhibited an enrichment of Pseudomonadota (phylum), Gammaproteobacteria (class), Enterobacterales (order), and Enterobacteriaceae (family), along with genera such as *Klebsiella*, *Shigella*, and *Escherichia*, as well as species like *Shigella sonnei*, and *Escherichia coli*. In contrast, the control group showed higher abundances of Succinivibrionaceae (family), Aeromonadales (order), and *Phocaeicola plebeius* (species), suggesting distinct microbial signatures associated with osteoporosis.

Analysis of microbial associations at both phylum, genus, and family levels with disease status revealed that while multiple taxa exhibited nominally significant associations in primary analyses (uncorrected *P* < 0.05), none of these associations remained statistically significant after false discovery rate (FDR) correction (all FDR-adjusted *P* > 0.05) (Supplementary Table 3).

### 3.4 The association between dietary pattern and gut microbiota

In the analysis investigating the association between the “animal offal and seeds” dietary pattern and gut microbiota composition, multivariable linear regression models (adjusted for age, sex, and other covariates) initially identified seven microbial taxa at the phylum level showing nominally significant associations (*P* < 0.05). Among these, the well-characterized phylum Actinomycetota demonstrated a negative association (β = −0.17, 95% CI: −0.32 to −0.03, *P* = 0.022). However, none of these associations remained statistically significant after false discovery rate (FDR) correction (all FDR-adjusted *P* > 0.05), a pattern similarly observed at the family level. At the genus level, three microbial taxa maintained statistically significant associations with the “animal offal and seeds” dietary pattern after false discovery rate (FDR) correction: Gemmatimonas showed the strongest positive association (β = 0.46, 95% CI: 0.32 to 0.59, *P*_*FDR*_ < 0.001), followed by Tindallia (β = 0.32, 95% CI: 0.18 to 0.46, *P*_*FDR*_ = 0.015) and Coprobacter (β = 0.30, 95% CI: 0.16 to 0.45, *P*_*FDR*_ = 0.019) (Supplementary Table 4).

### 3.5 Association between plasma metabolite and osteoporosis

**FIGURE 4:**
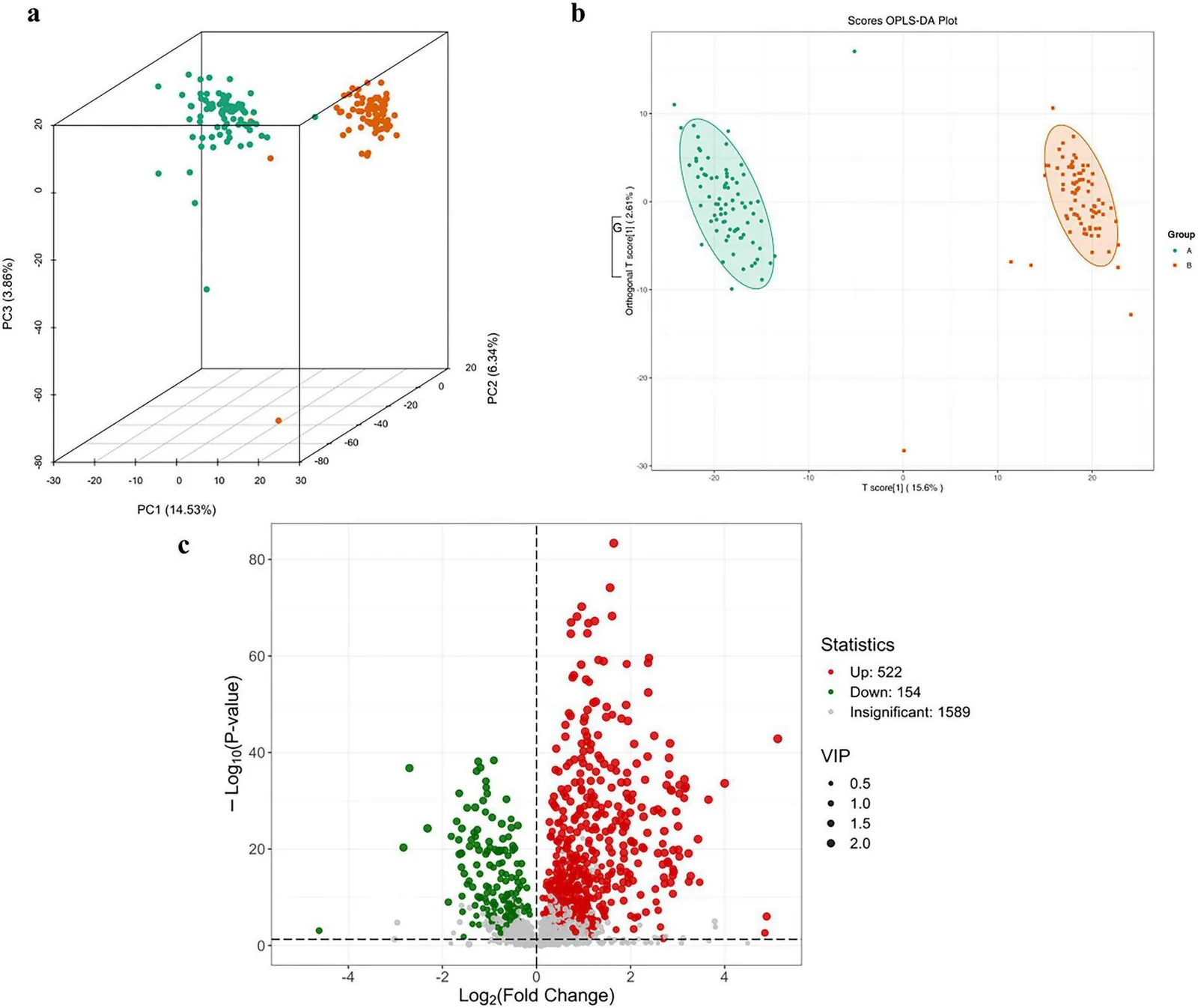
Metabolomics analysis. **(a)** Principal component analysis (PCA). **(b)** Orthogonal partial least squares discriminant analysis (OPLS-DA). **(c)** Volcano plot-demonstrate the differences in the relative amounts of metabolites in the two groups of samples and the statistical difference in significance of the difference.

Given the crucial role of gut microbiota in host metabolic regulation, plasma metabolome profiling provides a robust reflection of microbial functional activity. Untargeted metabolomic analysis identified 2,546 plasma metabolites. Initial partial least squares-discriminant analysis (PLS-DA) revealed significant metabolic differences between groups (Figure 4a), which were further confirmed by orthogonal PLS-DA (OPLS-DA) showing distinct clustering between osteoporosis (OP) patients and controls (Figure 4b). The OPLS-DA model demonstrated excellent performance, with high goodness-of-fit (R^2^Y = 0.978) and predictive ability (Q^2^ = 0.943, *P* < 0.05). Comparative metabolomic analysis identified 674 differentially abundant metabolites (VIP > 1, *P* < 0.05), comprising 522 upregulated and 152 downregulated species in OP patients compared to controls (Figure 4c).

KEGG pathway enrichment analysis of differential metabolites revealed significant enrichment (*P*_*FDR*_ < 0.05) in the following metabolic pathways: glyoxylate and dicarboxylate metabolism, carbon metabolism, amino acid biosynthesis, nicotinate and nicotinamide metabolism, alanine/aspartate/glutamate metabolism, cofactor biosynthesis, butanoate metabolism, glycine/serine/threonine metabolism, 2-oxocarboxylic acid metabolism, protein digestion and absorption, sulfur metabolism, pentose and glucuronate interconversions, valine/leucine/isoleucine biosynthesis, phosphate and phosphonate metabolism, lipoic acid metabolism, nucleotide metabolism, and aminoacyl-tRNA biosynthesis (complete list in Supplementary Table 5).

In the fully adjusted binary logistic regression model, comprehensive metabolomic profiling identified 979 plasma metabolites significantly associated with osteoporosis risk (*P*_*FDR*_ < 0.05).

### 3.6 Association between dietary pattern and plasma metabolite

To investigate diet-metabolite relationships, 56 metabolites were identified as significantly associated with the “animal offal and seeds” dietary pattern at the same significance threshold (*P*_*FDR*_ < 0.05; Supplementary Table 6). Through KEGG pathway annotation analysis, the 56 diet-associated metabolites were mainly mapped to five core metabolic modules: glycerophospholipid metabolism, arachidonic acid metabolism, linoleic acid metabolism, α-linolenic acid metabolism, and steroid hormone biosynthesis.

### 3.7 Mediating role of metabolomic markers between dietary pattern and osteoporosis

**FIGURE 5:**
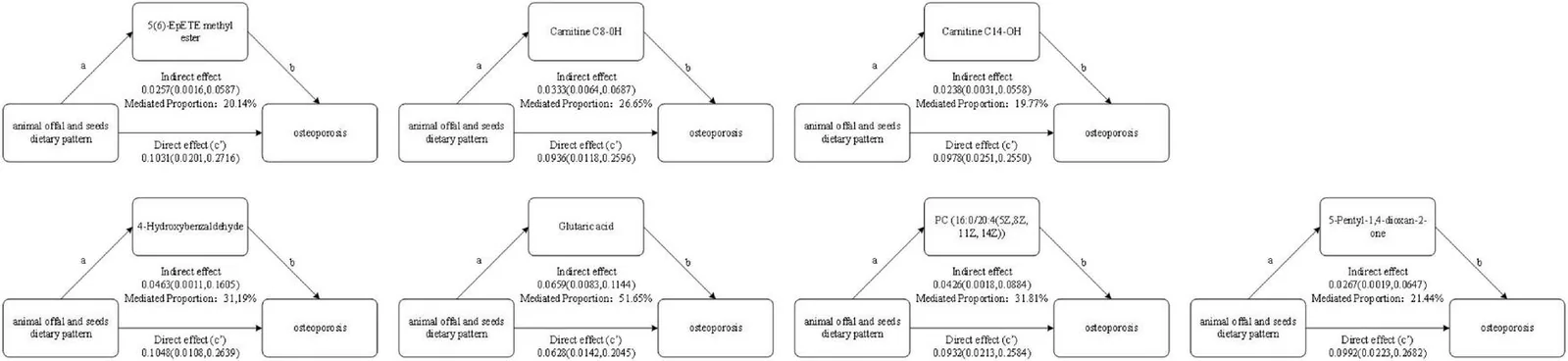
Mediation analysis of metabolomic markers between dietary pattern and osteoporosis.

Mediation analysis was conducted to identify plasma metabolites that significantly mediated the association between the “animal offal and seeds” dietary pattern and osteoporosis risk (selection threshold: *P*_*FDR*_ < 0.05). Among the 28 mediation models constructed, seven key metabolomic mediators were identified (Figure 5). These mediators included: 5(6)-EpETE methyl ester, Carnitine C8-0H, Carnitine C14-OH, 4-Hydroxybenzaldehyde, Glutaric acid, PC [16:0/20:4 (52,82, 11Z, 14Z)] and 5-Pentyl-1,4-dioxan-2-one.

## 4 Discussion

### 4.1 Main findings

This case-control study systematically examined the association between dietary patterns and osteoporosis among high-altitude population. Key observations showed that the “animal offal and seeds” dietary pattern had an independent association with osteoporosis. Gut microbiome profiling identified significantly higher microbial α-diversity (Simpson index, *P* < 0.05) in osteoporosis cases, accompanied by the enrichment of pathogenic Enterobacteriaceae taxa. Distinct plasma metabolomic signatures were observed between the case and control groups. Mediation analysis screened seven pivotal metabolites that acted as mediators for the diet-osteoporosis association: 5(6)-EpETE methyl ester, Carnitine C8-OH, Carnitine C14-OH, 4-Hydroxybenzaldehyde, Glutaric acid, PC [16:0/20:4 (5Z,8Z, 11Z, 14Z)] and 5-Pentyl-1,4-dioxan-2-one.

### 4.2 Dietary pattern and osteoporosis risk

This study employed principal component analysis (PCA) to assess dietary patterns and their associations with osteoporosis risk in high-altitude population. After comprehensive adjustment for confounders, only the “animal offal and seeds” dietary pattern demonstrated a significant positive association with osteoporosis risk (characterized by high factor loadings: animal offal = 0.637, seeds = 0.787). Compared to plain regions, the dietary structure in high-altitude areas is relatively monotonous, primarily consisting of highland barley and beef-lamb products, which is consistent with the dietary pattern extracted in the present research.

Several plausible mechanisms may explain the linkage between this dietary profile and altered bone-related status. First, animal offal is abundant in retinol, and the high-fat seeds promote the intestinal absorption of fat-soluble vitamin A; excessive retinol intake is capable of inhibiting bone formation and accelerating bone resorption (16–18). Second, the saturated fat and cholesterol from animal offal, combined with seeds dominated by ω-6-rich varieties such as sunflower seeds and processed peanuts, raise the pro-inflammatory potential of the whole diet and lack the bone-protective effects of ω-3 polyunsaturated fatty acids (19). Third, iron and zinc derived from animal offal compete with calcium and magnesium from seeds for intestinal absorption sites, and frequent large-volume intake under this dietary pattern magnifies mineral absorption antagonism. Fourth, both animal offal and seeds belong to high-energy-dense foods. Long-term adherence to this eating habit easily causes excess energy intake, metabolic disorders and chronic low-grade inflammation. When combined with high-altitude hypoxia, elevated oxidative stress in the bone microenvironment may activate osteoclasts and inhibit osteoblast activity.

Apart from the above-mentioned nutrient-related pathways, the high-fat, high-cholesterol and low-fiber features of the “animal offal and seeds” pattern may also impair calcium absorption, induce oxidative stress and provoke chronic inflammation (20, 21). Insufficient dietary-fiber intake can lower gut-microbiota-derived short-chain fatty-acid (SCFA) yield and further modify the bioavailability of phytic-acid-bound calcium and magnesium (22–24). These observational associations offer a reference for dietary research, and reasonable regulation on the intake of this dietary pattern may contribute to osteoporosis-related risk management among high-altitude inhabitants.

### 4.3 Metabolites and osteoporosis

Non-targeted metabolomics revealed significantly enriched metabolic pathways of differential metabolites between the two groups, with pathways closely related to bone metabolism, including purine metabolism, tryptophan metabolism, arginine and proline metabolism, sphingolipid metabolism, steroid biosynthesis, primary bile acid biosynthesis, glutathione metabolism, glycolysis/gluconeogenesis, glycerophospholipid metabolism, and retinol metabolism. One study demonstrated that the tryptophan metabolite melatonin, produced by gut microbiota, can alleviate clinical symptoms associated with osteoporosis and reverse gut dysbiosis. Melatonin exerts this effect by increasing the production of short-chain fatty acids and reducing TMAO, while also regulating macrophage homeostasis and lowering pro-inflammatory cytokine levels (25). A core metabolite in the sphingolipid metabolism pathway is sphingosine-1-phosphate (S1P), whose production and degradation are entirely dependent on the sphingolipid metabolic network. S1P acts as an osteoclast-osteoblast coupling factor, promoting osteoblast proliferation and bone formation. The recruitment of osteoclast precursors to bone resorption sites is regulated by the interaction between S1P gradients and S1P receptor expression (26). Additionally, a longitudinal multi-omics study validated these findings, showing that sphingosine-1-phosphate levels increased in a Han Chinese population after high-altitude exposure, approaching levels observed in Tibetan populations (27). In this study, when linking osteoporosis-related metabolites with the “animal offal and seeds” dietary pattern, seven metabolic markers were found to mediate the association between the dietary pattern and osteoporosis. The abnormal accumulation of Carnitine C8-OH and C14-OH reflects disrupted fatty acid β-oxidation, while glutaric acid directly inhibits key enzymes in the tricarboxylic acid (TCA) cycle. These changes collectively lead to insufficient energy supply for osteoblasts and excessive reactive oxygen species (ROS) production, ultimately impairing bone formation capacity (28). 5(6)-EpETE methyl ester and PC (16:0/20:4), as metabolites of arachidonic acid, suggest that excessive dietary ω-6 polyunsaturated fatty acids may promote eicosanoid production, activate inflammatory responses, and stimulate osteoclast activity (29). 5-Pentyl-1,4-dioxan-2-one, likely derived from gut microbial metabolism of dietary components, supports the importance of the “gut-bone axis” in osteoporosis pathogenesis. These findings suggest that high-altitude osteoporosis may involve dysregulated inflammatory responses, impaired energy metabolism, abnormal cholesterol metabolism, and gut microbiota dysbiosis, with dietary lipids potentially serving as key drivers of these pathways.

### 4.4 Enterobacteriaceae: gut characteristics of high-altitude osteoporosis

Consistent with previous findings (30) demonstrating increased gut microbial diversity in individuals with low bone mineral density, our study similarly revealed higher Simpson index values in osteoporosis patients compared to controls. Bacteroidota, major producers of short-chain fatty acids (SCFAs), may protect against bone loss through anti-inflammatory mechanisms, while Firmicutes may indirectly impair bone metabolism by promoting fat absorption and inflammation. Notably, the Firmicutes/Bacteroidetes (F/B) ratio was elevated in the osteoporosis group, with a higher F/B ratio indicating an aggravated inflammatory status. LEfSe analysis identified nine differentially enriched microbial biomarkers in the osteoporosis group, predominantly centered around the family Enterobacteriaceae. These facultative anaerobic potential pathogens may exacerbate bone metabolism dysregulation through multiple mechanisms. For example, Enterobacteriaceae (e.g., *Klebsiella*, *Escherichia*) may exacerbate bone loss by activating TLR4/NF-κB-mediated inflammation (e.g., IL-6, TNF-α), which promotes osteoclastogenesis and impairs osteoblast activity (31, 32). In contrast, the control group harbored beneficial taxa like succinate-producing Succinivibrionaceae and SCFA-producing *P. plebeius*, which enhance gut barrier integrity and mineral absorption while suppressing inflammation (33, 34). This contrasts with low-altitude osteoporosis studies, where Lactobacillaceae/Bifidobacterium depletion is more common (35, 36). The divergence may stem from high-altitude-specific factors: a high-fat/low-fiber diet (“animal offal and seeds” dietary pattern) and hypoxia likely synergize to promote Enterobacteriaceae overgrowth while suppressing SCFA producers, disrupting the gut-bone axis. Future studies should clarify whether Enterobacteriaceae enrichment is a high-altitude-specific driver or a universal biomarker of bone metabolism dysregulation, and validate the efficacy of dietary interventions (e.g., fiber supplementation) in improving bone health among high-altitude population.

Accumulating reviews have elaborated the close-knit association between gut microbiota-derived metabolites and bone homeostasis via the gut-bone axis, and multi-layered crosstalk of this axis participates in the onset and progression of osteoporosis (37, 38). In our high-altitude cohort, enriched Enterobacteriaceae may alter lysine metabolism and glutaric-acid-related mitochondrial energy metabolism, as well as trigger chronic low-grade inflammation, which serves as one set of novel gut-bone-axis mechanisms under plateau hypoxia and characteristic high-fat-low-fiber dietary habits.

This study reveals characteristic alterations in the gut microbiota of osteoporosis patients in high-altitude regions, with distinct community structural features that significantly differ from previously reported patterns in low-altitude population, which are typically characterized by reduced abundances of Lactobacillus and Bifidobacterium. Integrated metabolomics analysis suggests that Enterobacteriaceae may contribute to bone metabolism dysregulation through two mechanisms: first, its unique lysine degradation pathway promotes glutaric acid accumulation, thereby inhibiting mitochondrial energy metabolism; second, the chronic low-grade inflammatory state it induces further disrupts the dynamic balance of bone remodeling. Based on these findings, future research should focus on two key directions: (1) systematically validating the mechanistic effects of Enterobacteriaceae on glutamate metabolic pathways and bone microstructure using germ-free animal models; and (2) developing targeted intervention strategies (e.g., specific dietary modulation) to provide novel insights for the precise prevention and treatment of osteoporosis in high-altitude regions.

### 4.5 Strengths and limitations

This study has several strengths: (1) As a case-control study in a high-altitude population, it provides unique regional and ethnic insights into the dietary patterns, gut microbiota/metabolites, and osteoporosis. (2) The study establishes an integrative framework combining dietary pattern analysis with plasma metabolomics and gut microbiome profiling, offering multidimensional insights into osteoporosis in high-altitude population. However, there are limitations: (1) The case-control design precludes causal inferences; prospective cohort or interventional studies are needed. (2) Due to the constraints of medical infrastructure in the high-altitude study region, bone density measurements were obtained through quantitative ultrasound instead of the gold-standard dual-energy X-ray absorptiometry (DXA). While previous studies have demonstrated moderate correlation between quantitative ultrasound and DXA (39, 40), future investigations should incorporate DXA measurements for definitive osteoporosis diagnosis.

## 5 Conclusion

This study demonstrates that adherence to an “animal offal and seeds” dietary pattern is significantly associated with elevated osteoporosis risk among high-altitude population, with plasma metabolite playing a pivotal mediating role in this dietary-bone health relationship. Importantly, distinct gut microbial dysbiosis was observed in osteoporosis patients, most notably characterized by increased Enterobacteriaceae abundance. Through comprehensive metabolomic analysis, several key metabolite mediators were identified - including glutaric acid, and specific acylcarnitine species - that may mechanistically underlie the detrimental effects of this dietary pattern on bone metabolism. These findings provide novel insights into the metabolic pathways through which high-altitude dietary habits may contribute to osteoporosis pathogenesis.

## Data Availability

The datasets generated and/or analysed during the current study are not publicly available due to the protection of participant privacy and ethical restrictions, but are available from the corresponding author upon reasonable request.
