# Supplementary material for: Dietary patterns and osteoporosis risk in a high-altitude population: the roles of gut microbiota and plasma metabolites in a case-control study
Source: Front Nutr. 2026 Sep 10;13:1865666. doi: 10.3389/fnut.2026.1865666 (PMC13600886; doi:10.3389/fnut.2026.1865666)
Supplement: Supplementary file 1 [file Table_1.docx]

**Dietary Patterns and Osteoporosis Risk in High-Altitude Populations: The Roles of Gut Microbiota and Plasma Metabolites in a Case-Control Study. Luhan Zhang et al.**

**Supplemental Figure 1. Technical roadmap**

**Supplemental Table 1. Factor Loadings in Factor Analysis.**

| Food Groups | Factor1 | Factor2 | Factor3 | Factor4 | Factor5 | Factor6 |
| --- | --- | --- | --- | --- | --- | --- |
| Refined Grains | -0.117 | -0.051 | **0.905** | -0.21 | -0.033 | -0.096 |
| Whole Grains and Legumes | -0.456 | -0.346 | **-0.599** | -0.066 | -0.232 | 0.008 |
| Tubers | 0.416 | -0.082 | 0.304 | 0.301 | -0.11 | -0.112 |
| Beans and Bean Products | **0.662** | 0.214 | -0.052 | -0.267 | -0.114 | -0.183 |
| Vegetables | **0.783** | 0.244 | 0.006 | 0.135 | 0.004 | -0.061 |
| Mushroom and Seaweed | 0.051 | **0.74** | 0.121 | 0.256 | -0.096 | 0.035 |
| Fruits | **0.549** | -0.12 | 0.16 | -0.099 | 0.093 | 0.147 |
| Dairy Products | -0.113 | -0.125 | -0.381 | -0.192 | **0.511** | -0.327 |
| Red Meat | -0.069 | 0.027 | -0.104 | -0.055 | 0.053 | **0.896** |
| Poultry | 0.073 | **0.839** | -0.023 | -0.007 | 0.045 | 0.007 |
| Aquatic Products | 0.162 | **0.903** | -0.029 | -0.119 | -0.066 | 0.006 |
| Eggs | **0.743** | 0.126 | -0.083 | -0.049 | 0.054 | 0.002 |
| Animal Offal | -0.129 | 0.081 | -0.177 | **0.637** | 0.202 | -0.082 |
| Nuts and Seeds | 0.01 | 0.026 | 0.018 | **0.787** | -0.194 | 0.027 |
| Salt | -0.046 | -0.006 | 0.111 | -0.078 | **0.722** | 0.374 |
| Oils | 0.453 | -0.067 | 0.021 | 0.195 | **0.592** | -0.169 |

Note: Factor1, Plant-based Diet; Factor2, High-Protein Seafood & Poultry Diet; Factor3, Refined Grain-Dominant Diet; Factor4, Animal offal and seeds Diet; Factor5, High-Salt & High-Oil Diet; Factor6, Red Meat-Based Diet.

**Supplemental Table 2. Results of LEfSe analyses of species differing between groups**

| Taxonomy | Abundace | LDA_  score | *P* |
| --- | --- | --- | --- |
| k__Bacteria;p__Pseudomonadota | 4.84 | 4.20 | 0.021 |
| k__Bacteria;p__Pseudomonadota;c__Gammaproteobacteria | 4.76 | 4.24 | 0.008 |
| k__Bacteria;p__Pseudomonadota;c__Gammaproteobacteria;o__Enterobacterales | 4.67 | 4.21 | 0.005 |
| k__Bacteria;p__Pseudomonadota;c__Gammaproteobacteria;o__Aeromonadales | 3.85 | 3.09 | 0.003 |
| k__Bacteria;p__Pseudomonadota;c__Gammaproteobacteria;o__Enterobacterales;f__Enterobacteriaceae | 4.66 | 4.21 | 0.003 |
| k__Bacteria;p__Pseudomonadota;c__Gammaproteobacteria;o__Aeromonadales;f__Succinivibrionaceae | 3.84 | 3.08 | 0.008 |
| k__Bacteria;p__Pseudomonadota;c__Gammaproteobacteria;o__Enterobacterales;f__Enterobacteriaceae;g__Escherichia | 4.38 | 3.97 | 0.001 |
| k__Bacteria;p__Pseudomonadota;c__Gammaproteobacteria;o__Enterobacterales;f__Enterobacteriaceae;g__Shigella | 3.59 | 3.19 | 0.004 |
| k__Bacteria;p__Pseudomonadota;c__Gammaproteobacteria;o__Enterobacterales;f__Enterobacteriaceae;g__Klebsiella | 3.72 | 3.15 | 0.020 |
| k__Bacteria;p__Pseudomonadota;c__Gammaproteobacteria;o__Enterobacterales;f__Enterobacteriaceae;g__Escherichia;s__Escherichia_coli | 4.35 | 3.88 | 0.002 |
| k__Bacteria;p__Bacteroidota;c__Bacteroidia;o__Bacteroidales;f__Bacteroidaceae;g__Phocaeicola;s__Phocaeicola_plebeius | 4.08 | 3.38 | 0.007 |
| k__Bacteria;p__Pseudomonadota;c__Gammaproteobacteria;o__Enterobacterales;f__Enterobacteriaceae;g__Shigella;s__Shigella_sonnei | 3.46 | 3.01 | 0.005 |

**Supplemental Table 3. Association of osteoporosis with gut microorganisms**

| Variable | *OR* | 95% *CI* | *P* | *P_FDR_* |
| --- | --- | --- | --- | --- |
| k__Bacteria;p__Pseudomonadota | 3.71 | (1.52, 11.15) | 0.011 | 0.972 |
| f__Chitinophagaceae;g__Flavihumibacter | 0.49 | (0.28, 0.77) | 0.004 | 0.995 |
| f__Enterobacteriaceae;g__Kluyvera | 3.61 | (1.60, 9.41) | 0.005 | 0.995 |
| f__Enterobacteriaceae;g__Cedecea | 3.59 | (1.62, 9.97) | 0.005 | 0.995 |
| f__Erwiniaceae;g__Erwinia | 3.07 | (1.51, 7.87) | 0.007 | 0.995 |
| f__Enterobacteriaceae;g__Leclercia | 2.19 | (1.29, 4.22) | 0.008 | 0.995 |
| f__Xanthomonadaceae;g__Pseudoxanthomonas | 2.10 | (1.25, 3.97) | 0.009 | 0.995 |
| f__Enterobacteriaceae;g__Cronobacter | 2.54 | (1.38, 5.78) | 0.009 | 0.995 |
| f__Clostridiaceae;g__Tindallia | 1.83 | (1.17, 2.95) | 0.010 | 0.995 |
| f__Enterobacteriaceae;g__Yokenella | 2.39 | (1.32, 5.48) | 0.014 | 0.995 |
| f__Desulfococcaceae;g__Desulfococcus | 2.01 | (1.16, 3.93) | 0.021 | 0.995 |
| f__Enterobacteriaceae;g__Shigella | 4.73 | (1.60, 25.08) | 0.022 | 0.995 |
| f__Enterobacteriaceae;g__Escherichia | 4.36 | (1.52, 20.54) | 0.022 | 0.995 |
| f__Symbiobacteriaceae;g__Symbiobacterium | 3.36 | (1.28, 10.18) | 0.024 | 0.995 |
| f__Peptostreptococcaceae;g__Romboutsia | 0.24 | (0.06, 0.74) | 0.024 | 0.995 |
| f__Enterobacteriaceae;g__Salmonella | 2.75 | (1.27, 7.23) | 0.025 | 0.995 |
| f__Enterobacteriaceae;g__Kosakonia | 5.10 | (1.63, 27.83) | 0.025 | 0.995 |
| f__Bacillaceae;g__Thalassobacillus | 0.33 | (0.11, 0.81) | 0.026 | 0.995 |
| f__Comamonadaceae;g__Variovorax | 1.54 | (1.05, 2.34) | 0.030 | 0.995 |
| f__Hymenobacteraceae;g__Rufibacter | 1.95 | (1.14, 4.14) | 0.033 | 0.995 |
| f__Ectothiorhodospiraceae;g__Ectothiorhodospira | 2.10 | (1.16, 4.76) | 0.037 | 0.995 |
| f__Yersiniaceae;g__Rahnella | 1.74 | (1.07, 3.03) | 0.038 | 0.995 |
| f__Peptostreptococcaceae;g__Paeniclostridium | 0.53 | (0.27, 0.92) | 0.039 | 0.995 |
| f__Lachnospiraceae;g__Lachnoclostridium | 1.58 | (1.08, 2.67) | 0.040 | 0.995 |
| f__Methylococcaceae;g__Methylococcus | 3.79 | (1.41, 20.89) | 0.045 | 0.995 |
| f__Burkholderiaceae;g__Paraburkholderia | 0.68 | (0.46, 0.98) | 0.046 | 0.995 |
| f__Nosematidae;g__Nosema | 0.20 | (0.03, 0.82) | 0.048 | 0.995 |
| o__Enterobacterales;f__Enterobacteriaceae | 4.84 | (1.69, 19.36) | 0.011 | 0.998 |
| o__Enterobacterales;f__Yersiniaceae | 2.20 | (1.26, 4.41) | 0.012 | 0.998 |
| o__Enterobacterales;f__Erwiniaceae | 2.76 | (1.35, 7.75) | 0.020 | 0.998 |
| o__Candidatus_Brocadiales;f__Candidatus_Brocadiaceae | 0.58 | (0.35, 0.89) | 0.021 | 0.998 |
| o__Eubacteriales;f__Symbiobacteriaceae | 3.36 | (1.28, 10.18) | 0.024 | 0.998 |
| o__Chromatiales;f__Ectothiorhodospiraceae | 1.97 | (1.14, 3.86) | 0.026 | 0.998 |
| o__Desulfobacterales;f__Desulfococcaceae | 2.03 | (1.12, 4.04) | 0.028 | 0.998 |
| o__Other;f__Nosematidae | 0.20 | (0.03, 0.81) | 0.046 | 0.998 |

**Supplemental Table 4. Association of Nuts & Organ Meat Diet with gut microorganisms**

| Variable | *β* | 95% *CI* | *P* | *P_FDR_* |
| --- | --- | --- | --- | --- |
| k__Bacteria;p__Candidatus_Zambryskibacteria | 0.24 | (0.09, 0.39) | 0.002 | 0.296 |
| k__Bacteria;p__Candidatus_Saccharibacteria | -0.20 | (-0.34, -0.06) | 0.006 | 0.41 |
| k__Bacteria;p__Candidatus_Collierbacteria | 0.20 | (0.05, 0.35) | 0.011 | 0.48 |
| k__Bacteria;p__Actinomycetota | -0.17 | (-0.32, -0.03) | 0.022 | 0.594 |
| k__Bacteria;p__Candidatus_Moranbacteria | 0.18 | (0.02, 0.33) | 0.023 | 0.594 |
| k__Bacteria;p__Candidatus_Peregrinibacteria | 0.16 | (0.01, 0.32) | 0.038 | 0.761 |
| k__Bacteria;p__Bdellovibrionota | 0.15 | (0.01, 0.30) | 0.042 | 0.761 |
| f__Gemmatimonadaceae;g__Gemmatimonas | 0.46 | (0.32, 0.59) | <0.001 | <0.001 |
| f__Clostridiaceae;g__Tindallia | 0.32 | (0.18, 0.46) | <0.001 | 0.015 |
| f__Barnesiellaceae;g__Coprobacter | 0.30 | (0.16, 0.45) | <0.001 | 0.019 |
| f__Rikenellaceae;g__Rikenella | 0.28 | (0.13, 0.42) | <0.001 | 0.102 |
| f__Chromatiaceae;g__Rheinheimera | 0.27 | (0.12, 0.42) | 0.001 | 0.194 |
| f__Lactobacillaceae;g__Companilactobacillus | -0.24 | (-0.37, -0.10) | 0.001 | 0.211 |
| f__Promicromonosporaceae;g__Isoptericola | -0.24 | (-0.38, -0.10) | 0.001 | 0.229 |
| f__Lactobacillaceae;g__Fructilactobacillus | -0.23 | (-0.37, -0.09) | 0.001 | 0.229 |
| f__Geodermatophilaceae;g__Geodermatophilus | -0.24 | (-0.39, -0.09) | 0.002 | 0.290 |
| f__Veillonellaceae;g__Allisonella | -0.22 | (-0.36, -0.08) | 0.002 | 0.290 |
| f__Lactobacillaceae;g__Pediococcus | -0.22 | (-0.36, -0.08) | 0.002 | 0.318 |
| f__Tannerellaceae;g__Tannerella | 0.22 | (0.08, 0.37) | 0.002 | 0.318 |
| f__Rhodospirillaceae;g__Roseospira | 0.23 | (0.08, 0.38) | 0.003 | 0.380 |
| f__Desulfobacteraceae;g__Desulfotignum | 0.23 | (0.08, 0.38) | 0.003 | 0.380 |
| f__Rubrobacteraceae;g__Rubrobacter | -0.22 | (-0.37, -0.07) | 0.004 | 0.395 |
| f__Other;g__Candidatus_Minimicrobia | -0.21 | (-0.35, -0.07) | 0.004 | 0.422 |
| f__Weeksellaceae;g__Cloacibacterium | 0.21 | (0.07, 0.36) | 0.005 | 0.422 |
| f__Lachnospiraceae;g__Anaerobutyricum | -0.20 | (-0.34, -0.06) | 0.006 | 0.516 |
| f__Candidatus_Saccharimonadaceae;g__Candidatus_Saccharimonas | -0.20 | (-0.35, -0.06) | 0.006 | 0.516 |
| f__Thermaceae;g__Meiothermus | 0.20 | (0.06, 0.35) | 0.007 | 0.516 |
| f__Coriobacteriaceae;g__Collinsella | -0.20 | (-0.35, -0.06) | 0.007 | 0.516 |
| f__Arcobacteraceae;g__Arcobacter | 0.21 | (0.06, 0.36) | 0.007 | 0.516 |
| f__Xanthobacteraceae;g__Aquabacter | 0.21 | (0.06, 0.36) | 0.007 | 0.516 |
| f__Bogoriellaceae;g__Georgenia | -0.20 | (-0.34, -0.05) | 0.008 | 0.537 |
| f__Lachnospiraceae;g__Velocimicrobium | -0.20 | (-0.35, -0.05) | 0.009 | 0.573 |
| f__Comamonadaceae;g__Variovorax | 0.20 | (0.05, 0.34) | 0.010 | 0.599 |
| f__Crenotrichaceae;g__Crenothrix | 0.20 | (0.05, 0.35) | 0.010 | 0.618 |
| f__Reichenbachiellaceae;g__Marinoscillum | 0.19 | (0.04, 0.34) | 0.011 | 0.628 |
| f__Steigviridae;g__Kahnovirus | 0.20 | (0.04, 0.35) | 0.012 | 0.644 |
| f__Actinomycetaceae;g__Actinomyces | -0.19 | (-0.34, -0.04) | 0.014 | 0.729 |
| f__Halomonadaceae;g__Salinicola | 0.19 | (0.04, 0.34) | 0.016 | 0.814 |
| f__Suoliviridae;g__Burzaovirus | 0.18 | (0.03, 0.33) | 0.018 | 0.886 |
| f__Pectobacteriaceae;g__Pectobacterium | 0.18 | (0.03, 0.33) | 0.019 | 0.901 |
| f__Thermoanaerobacteraceae;g__Calorimonas | 0.18 | (0.03, 0.33) | 0.019 | 0.911 |
| f__Enterococcaceae;g__Enterococcus | -0.17 | (-0.31, -0.03) | 0.020 | 0.912 |
| f__Microbacteriaceae;g__Microbacterium | -0.17 | (-0.32, -0.02) | 0.025 | 0.998 |
| f__Flavobacteriaceae;g__Flavimarina | 0.17 | (0.02, 0.33) | 0.026 | 0.998 |
| f__Saccotheciaceae;g__Aureobasidium | -0.17 | (-0.31, -0.02) | 0.026 | 0.998 |
| f__Bacteroidaceae;g__Bacteroides | 0.16 | (0.02, 0.31) | 0.026 | 0.998 |
| f__Lactobacillaceae;g__Weissella | -0.16 | (-0.31, -0.02) | 0.028 | 0.998 |
| f__Pasteurellaceae;g__Bibersteinia | -0.16 | (-0.30, -0.02) | 0.030 | 0.998 |
| f__Odoribacteraceae;g__Odoribacter | 0.16 | (0.01, 0.31) | 0.038 | 0.998 |
| f__Intrasporangiaceae;g__Tetrasphaera | -0.16 | (-0.31, -0.01) | 0.040 | 0.998 |
| f__Bifidobacteriaceae;g__Bifidobacterium | -0.15 | (-0.30, -0.01) | 0.042 | 0.998 |
| f__Bacillaceae;g__Calidifontibacillus | 0.16 | (0.00, 0.31) | 0.043 | 0.998 |
| f__Terasakiellaceae;g__Terasakiella | 0.14 | (0.00, 0.28) | 0.044 | 0.998 |
| f__Bifidobacteriaceae;g__Gardnerella | -0.15 | (-0.30, 0.00) | 0.045 | 0.998 |
| f__Nitrospinaceae;g__Nitrospina | 0.15 | (0.00, 0.30) | 0.047 | 0.998 |
| f__Mycobacteriaceae;g__Mycobacteroides | -0.15 | (-0.30, 0.00) | 0.049 | 0.998 |
| f__Acidaminococcaceae;g__Succinispira | 0.15 | (0.00, 0.30) | 0.049 | 0.998 |
| o__Rubrobacterales;f__Rubrobacteraceae | -0.20 | (-0.35, -0.05) | 0.008 | 0.990 |
| o__Coriobacteriales;f__Coriobacteriaceae | -0.20 | (-0.35, -0.05) | 0.008 | 0.990 |
| o__Micrococcales;f__Bogoriellaceae | -0.20 | (-0.34, -0.05) | 0.008 | 0.990 |
| o__Methylococcales;f__Crenotrichaceae | 0.20 | (0.05, 0.35) | 0.010 | 0.990 |
| o__Actinomycetales;f__Actinomycetaceae | -0.18 | (-0.33, -0.03) | 0.017 | 0.990 |
| o__Desulfovibrionales;f__Desulfohalobiaceae | 0.18 | (0.03, 0.34) | 0.019 | 0.990 |
| o__Lactobacillales;f__Enterococcaceae | -0.17 | (-0.31, -0.03) | 0.021 | 0.990 |
| o__Bacteroidales;f__Barnesiellaceae | 0.18 | (0.02, 0.33) | 0.024 | 0.990 |
| o__Dothideales;f__Saccotheciaceae | -0.17 | (-0.31, -0.02) | 0.026 | 0.990 |
| o__Crassvirales;f__Steigviridae | 0.17 | (0.01, 0.32) | 0.034 | 0.990 |
| o__Micrococcales;f__Intrasporangiaceae | -0.16 | (-0.31, -0.01) | 0.038 | 0.990 |
| o__Cytophagales;f__Reichenbachiellaceae | 0.16 | (0.01, 0.31) | 0.038 | 0.990 |
| o__Other;f__Herelleviridae | 0.16 | (0.01, 0.31) | 0.040 | 0.990 |
| o__Micrococcales;f__Micrococcaceae | -0.15 | (-0.30, -0.01) | 0.040 | 0.990 |
| o__Bifidobacteriales;f__Bifidobacteriaceae | -0.15 | (-0.30, -0.01) | 0.042 | 0.990 |
| o__Rhodospirillales;f__Terasakiellaceae | 0.14 | (0.00, 0.28) | 0.044 | 0.990 |

**Supplemental Table 5. Enrichment of the KGEE metabolic pathway**

| Name | *P* | *P_FDR_* | Fold Enrichment | hitCompound |
| --- | --- | --- | --- | --- |
| Pentose phosphate pathway | <0.001 | <0.001 | 125.36 | D-Glyceraldehyde 3-phosphate,D-Erythrose 4-phosphate |
| Nicotinate and nicotinamide metabolism | <0.001 | <0.001 | 120.63 | 1-Methylnicotinamide |
| Drug metabolism - cytochrome P450 | <0.001 | <0.001 | 39.99 | 4-Hydroxycyclophosphamide,Codeine-6-glucuronide,3-Hydroxyvalproic acid |
| Purine metabolism | <0.001 | <0.001 | 63.06 | Xanthine,L-Glutamine,Xanthosine,IDP,Hypoxanthine,Inosine,Adenine |
| Tryptophan metabolism | <0.001 | <0.001 | 62.88 | L-Tryptophan,Melatonin,Serotonin,5-Hydroxykynurenine,5-Hydroxy-L-tryptophan,L-Kynurenine,Tryptamine,Indolepyruvate,5-Methoxyindoleacetate,N-Methyltryptamine |
| Pyrimidine metabolism | <0.001 | <0.001 | 74.11 | L-Glutamine,Uridine,CDP,dUMP,Deoxyuridine,Thymidine,2'-Deoxy-5-hydroxymethylcytidine-5'-diphosphate,Uracil |
| Glyoxylate and dicarboxylate metabolism | <0.001 | <0.001 | 69.09 | L-Serine,Glycine,L-Glutamine |
| Alanine, aspartate and glutamate metabolism | <0.001 | <0.001 | 66.55 | L-Asparagine,L-Alanine,L-Glutamine |
| D-Glutamine and D-glutamate metabolism | <0.001 | <0.001 | 77.63 | L-Glutamine |
| Nitrogen metabolism | <0.001 | <0.001 | 77.63 | L-Glutamine |
| Fatty acid biosynthesis | <0.001 | <0.001 | 77.38 | Dodecanoic acid |
| Arginine biosynthesis | <0.001 | <0.001 | 59.16 | L-Arginine,L-Glutamine |
| Arginine and proline metabolism | <0.001 | <0.001 | 59.60 | L-Arginine,L-Proline,Phosphocreatine |
| Sphingolipid metabolism | <0.001 | <0.001 | 43.90 | Sphingosine 1-phosphate,Sphinganine 1-phosphate,L-Serine,Phytosphingosine |
| Glycerolipid metabolism | <0.001 | <0.001 | 43.47 | Glycerol |
| Galactose metabolism | <0.001 | <0.001 | 22.08 | D-Glucose,D-Fructose,Galactitol,Glycerol,D-Mannose |
| Glycolysis / Gluconeogenesis | <0.001 | <0.001 | 34.65 | D-Glyceraldehyde 3-phosphate |
| Inositol phosphate metabolism | <0.001 | <0.001 | 34.65 | D-Glyceraldehyde 3-phosphate |
| Valine, leucine and isoleucine biosynthesis | <0.001 | <0.001 | 33.14 | L-Threonine,L-Isoleucine,L-Valine |
| Valine, leucine and isoleucine degradation | <0.001 | <0.001 | 34.14 | L-Valine,L-Isoleucine |
| Pantothenate and CoA biosynthesis | <0.001 | <0.001 | 34.01 | Pantetheine,L-Valine,Uracil |
| Glutathione metabolism | <0.001 | <0.001 | 33.45 | Glycine |
| Taurine and hypotaurine metabolism | <0.001 | <0.001 | 31.69 | Taurine |
| Aminoacyl-tRNA biosynthesis | <0.001 | <0.001 | 22.81 | L-Asparagine,L-Histidine,L-Phenylalanine,L-Arginine,L-Glutamine,Glycine,L-Serine,L-Methionine,L-Valine,L-Alanine,L-Lysine,L-Isoleucine,L-Threonine,L-Tryptophan,L-Tyrosine,L-Proline |
| Steroid biosynthesis | <0.001 | <0.001 | 11.22 | Calcidiol,Episterol,Cholesterol,3-Keto-4-methylzymosterol,Calcitriol,Desmosterol |
| Fatty acid degradation | <0.001 | <0.001 | 21.72 | L-Palmitoylcarnitine |
| Tyrosine metabolism | <0.001 | <0.001 | 21.10 | Dopamine,L-Tyrosine,4-Hydroxyphenylacetaldehyde,Thyroxine |
| Phenylalanine, tyrosine and tryptophan biosynthesis | <0.001 | <0.001 | 21.09 | L-Phenylalanine,L-Tyrosine |
| Phenylalanine metabolism | <0.001 | <0.001 | 21.08 | L-Phenylalanine,Phenylacetic acid,L-Tyrosine |
| Ubiquinone and other terpenoid-quinone biosynthesis | <0.001 | <0.001 | 21.10 | L-Tyrosine |
| Cysteine and methionine metabolism | <0.001 | <0.001 | 17.70 | L-Serine,L-Methionine |
| Pentose and glucuronate interconversions | <0.001 | <0.001 | 17.46 | D-Xylose |
| Porphyrin and chlorophyll metabolism | <0.001 | <0.001 | 11.76 | Glycine,Biliverdin,Bilirubin |
| Neomycin, kanamycin and gentamicin biosynthesis | <0.001 | <0.001 | 14.62 | D-Glucose |
| Primary bile acid biosynthesis | <0.001 | <0.001 | 9.72 | Cholesterol,Glycine,Taurine,Cholic acid |
| Biotin metabolism | <0.001 | <0.001 | 13.44 | Biotin,L-Lysine |
| Lysine degradation | <0.001 | <0.001 | 13.53 | L-Lysine |
| Selenocompound metabolism | <0.001 | 0.001 | 12.01 | L-Alanine |
| Starch and sucrose metabolism | 0.001 | 0.002 | 9.87 | D-Fructose,D-Glucose |
| Fructose and mannose metabolism | 0.001 | 0.002 | 8.72 | D-Fructose,D-Mannose,D-Glyceraldehyde 3-phosphate |
| Glycine, serine and threonine metabolism | 0.004 | 0.005 | 8.14 | L-Serine,Choline,Glycine,Sarcosine,L-Threonine |
| Glycerophospholipid metabolism | 0.004 | 0.005 | 8.06 | Choline,Acetylcholine |
| D-Arginine and D-ornithine metabolism | 0.004 | 0.005 | 7.96 | D-Ornithine |
| Retinol metabolism | 0.042 | 0.049 | 4.03 | Retinol,Retinyl ester |
| beta-Alanine metabolism | 0.043 | 0.049 | 4.05 | Uracil,L-Histidine |
| Histidine metabolism | 0.044 | 0.049 | 4.05 | L-Histidine |

**Supplemental Table 6. Relationship between animal visceral melon dietary patterns (factor 4) and plasma metabolomic markers**

| Variable | Coefficient | 95% *CI* | *P* | *P_FDR_* |
| --- | --- | --- | --- | --- |
| Cucurbitacin I | 0.51 | (0.36, 0.66) | <0.001 | <0.001 |
| Yohimbine | 0.49 | (0.34, 0.64) | <0.001 | <0.001 |
| 4-Nitrosobiphenyl | 0.39 | (0.26, 0.53) | <0.001 | <0.001 |
| Succinic acid semialdehyde | 0.38 | (0.23, 0.52) | <0.001 | <0.001 |
| PC(16:0/20:4(5Z,8Z,11Z,14Z)) | 0.39 | (0.24, 0.53) | <0.001 | <0.001 |
| 1-hexadecanoyl-2-(11Z-octadecenoyl)-sn-glycero-3-phosphocholine | 0.38 | (0.23, 0.53) | <0.001 | 0.001 |
| 16-Dehydroprogesterone | 0.37 | (0.22, 0.52) | <0.001 | 0.001 |
| 2,5-Dimethyloxazole | 0.33 | (0.19, 0.46) | <0.001 | 0.001 |
| 4-Hydroxybenzaldehyde | 0.36 | (0.21, 0.52) | <0.001 | 0.003 |
| PE-NMe2(16:0/20:2(11Z,14Z)) | 0.35 | (0.19, 0.50) | <0.001 | 0.004 |
| Carnitine C12-OH | 0.33 | (0.18, 0.47) | <0.001 | 0.005 |
| Carnitine C8-OH | 0.33 | (0.18, 0.48) | <0.001 | 0.009 |
| 6-trans-12-epi Leukotriene B4 | -0.30 | (-0.45, -0.16) | <0.001 | 0.010 |
| Pyridine-3,4-dicarboxylic acid | 0.31 | (0.16, 0.46) | <0.001 | 0.010 |
| Triethylene glycol | 0.28 | (0.14, 0.42) | <0.001 | 0.014 |
| Tapentadol | 0.32 | (0.16, 0.47) | <0.001 | 0.016 |
| Androstenedione | 0.31 | (0.15, 0.46) | <0.001 | 0.016 |
| 5(6)-EpETE methyl ester | 0.31 | (0.15, 0.47) | <0.001 | 0.019 |
| 1,11-Undecanedicarboxylic acid | 0.28 | (0.13, 0.42) | <0.001 | 0.023 |
| 3,5-Dichlorobiphenyl | 0.26 | (0.13, 0.40) | <0.001 | 0.023 |
| Resorcinol sulfoxide | 0.27 | (0.13, 0.42) | <0.001 | 0.023 |
| Ethyl 3-cyclohexyl-3-oxopropanoate | 0.27 | (0.13, 0.41) | <0.001 | 0.023 |
| 5-Methyl-2-[(1R,6R)-3-methyl-6-(1-methylethenyl)-2-cyclohexen-1-yl]-1,3-benzenediol | 0.31 | (0.15, 0.47) | <0.001 | 0.023 |
| Barbituric acid | 0.26 | (0.12, 0.39) | <0.001 | 0.027 |
| Phosphocholine | 0.28 | (0.13, 0.43) | <0.001 | 0.027 |
| Formestane | 0.28 | (0.13, 0.43) | <0.001 | 0.028 |
| L-(-)-Sorbose | 0.25 | (0.12, 0.38) | <0.001 | 0.028 |
| 1-(11Z-eicosenoyl)-2-(9Z-hexadecenoyl)-glycero-3-phosphoethanolamine | 0.29 | (0.13, 0.44) | <0.001 | 0.028 |
| Methyldiphenylphosphine oxide | 0.27 | (0.13, 0.42) | <0.001 | 0.028 |
| 1'H-5alpha-Androst-2-eno[3,2-b]indol-17beta-ol | 0.28 | (0.13, 0.44) | <0.001 | 0.028 |
| DG(20:2(11Z,14Z)/20:4(8Z,11Z,14Z,17Z)/0:0) | 0.28 | (0.13, 0.43) | <0.001 | 0.030 |
| Phophatidylethanolamine(22:1/14:0) | 0.29 | (0.13, 0.44) | <0.001 | 0.030 |
| 19-Hydroxyandrost-4-ene-3,17-dione | 0.29 | (0.13, 0.45) | <0.001 | 0.034 |
| Val-Ala-Ala | 0.26 | (0.12, 0.40) | <0.001 | 0.034 |
| Diphthamide | 0.26 | (0.11, 0.40) | <0.001 | 0.034 |
| 7-Mercaptoheptanoic acid | 0.25 | (0.11, 0.39) | <0.001 | 0.034 |
| 5-Pentyl-1,4-dioxan-2-one | 0.26 | (0.12, 0.41) | 0.001 | 0.036 |
| Chlorprothixene | 0.27 | (0.12, 0.41) | 0.001 | 0.037 |
| Ethiolate | 0.25 | (0.11, 0.39) | 0.001 | 0.038 |
| Oleamide | 0.28 | (0.12, 0.43) | 0.001 | 0.038 |
| Acetylsalicylic acid | 0.24 | (0.10, 0.37) | 0.001 | 0.038 |
| Noracymethadol | 0.28 | (0.12, 0.43) | 0.001 | 0.041 |
| Carnitine C14-OH | 0.26 | (0.11, 0.41) | 0.001 | 0.041 |
| N-Acetylneuraminic Acid(SA) | 0.27 | (0.11, 0.42) | 0.001 | 0.042 |
| Indole-3-carboxylic acid | 0.25 | (0.11, 0.40) | 0.001 | 0.042 |
| 2,4-Dihydroxypyrimidine-5-carboxylic acid | 0.24 | (0.10, 0.38) | 0.001 | 0.043 |
| 6β-hydroxytestosterone | 0.25 | (0.10, 0.39) | 0.001 | 0.044 |
| 2,5-Furandimethanol | 0.24 | (0.10, 0.38) | 0.001 | 0.044 |
| 1,2-Dioctadecanoyl-sn-glycero-3-phospho-(1'-myo-inositol) | 0.27 | (0.11, 0.42) | 0.001 | 0.047 |
| Myosmine | 0.24 | (0.10, 0.38) | 0.001 | 0.047 |
| 1-(2,2-Difluoroethyl)piperazine | 0.25 | (0.10, 0.39) | 0.001 | 0.047 |
| D-Allose | 0.25 | (0.10, 0.39) | 0.001 | 0.047 |
| 15(S)-HETE-d8 | 0.26 | (0.11, 0.42) | 0.001 | 0.047 |
| Glutaric acid | 0.25 | (0.10, 0.40) | 0.001 | 0.047 |
| Procaterol hydrochloride hemihydrate | 0.24 | (0.10, 0.38) | 0.001 | 0.047 |
| (2S)-2-azaniumyl-4-(2-azaniumyl-3-hydroxypropoxy)butanoate | 0.25 | (0.10, 0.39) | 0.001 | 0.049 |
